# Incidentally detected microcystic serous cystadenoma of the pancreas with splenic invasion: a case report and literature review

**DOI:** 10.1259/bjrcr.20190109

**Published:** 2020-09-29

**Authors:** Fumiko Yagi, Hirotaka Akita, Akihisa Ueno, Kiminori Takano, Yohei Masugi, Michiie Sakamoto, Minoru Kitago, Masahiro Shinoda, Yuko Kitagawa, Kenji Toyama, Yohji Matsusaka, Hideki Yashiro, Shigeo Okuda, Masahiro Jinzaki

**Affiliations:** 1 Department of Diagnostic Radiology, Keio University School of Medicine, Shinjuku-ku, Tokyo, Japan; 2 Department of Pathology, Keio University School of Medicine, Shinjuku-ku, Tokyo, Japan; 3 Department of Surgery, Keio University School of Medicine, Shinjuku-ku, Tokyo, Japan; 4 Department of Surgery, Hiratsuka City Hospital, Kanagawa, Japan; 5 Department of Diagnostic Radiology, Hiratsuka City Hospital, Kanagawa, Japan

## Abstract

Serous cystic neoplasms are relatively uncommon and rarely possess malignant potential. We report a rare case of pancreatic serous cystadenoma with splenic invasion in a female in her 60s. Dynamic contrast-enhanced CT revealed a 3 cm mass in the tail of the pancreas. The lesion showed marked enhancement in the arterial phase on dynamic CT, which extended into the spleen. No cystic components were detected in the pancreatic mass on either magnetic resonance cholangiopancreatography or *T*_2_ weighted imaging. No metastasis or lymph node swelling was detected. Based on the hypervascularity of the tumour, the pre-operative diagnosis was pancreatic neuroendocrine tumour with splenic invasion. The patient underwent laparoscopic distal pancreatectomy with splenectomy. The pathological diagnosis was microcystic serous cystadenoma with locally aggressive features (infiltration into spleen, lymph nodes, and splenic vein). A few cases of pancreatic serous cystadenomas with splenic invasion have been reported; all were symptomatic, with diameters greater than approximately 9 cm. This is the first known case of incidentally detected serous cystadenoma with splenic invasion, reported with detailed imaging findings of dynamic CT and MRI.

## Clinical presentation

A female in her 60s, with no significant medical or family history, underwent an annual medical check-up at a different hospital and was diagnosed with diabetes mellitus. An abdominal ultrasonography screening revealed a hypoechoic mass in the tail of the pancreas. Physical examination, laboratory studies that included tumour markers (carcinoembryonic antigen, neuron-specific enolase, and carbohydrate antigen 19–9), and hormonal examination (insulin, glucagon, and gastrin) all revealed normal results. The patient was referred to our hospital for further examination and treatment.

## Image findings

**Figure 1.**
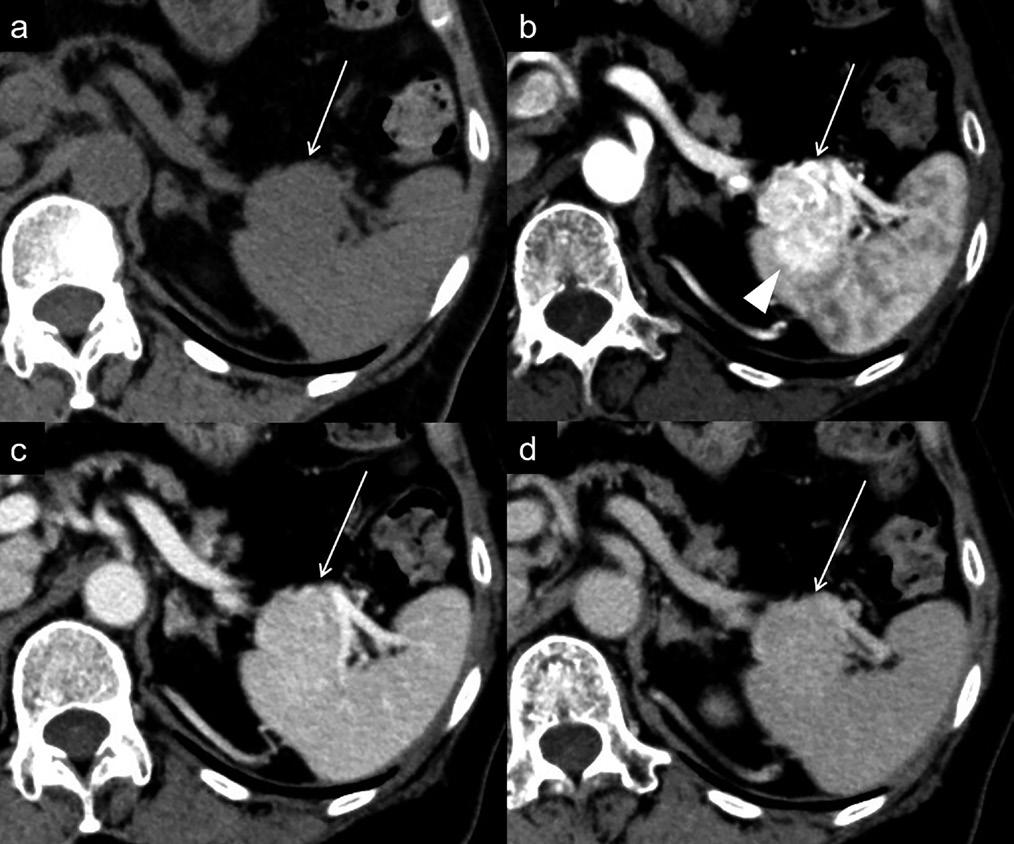
Dynamic contrast-enhanced CT (a. unenhanced phase, b. arterial phase, c. portal venous phase, d. equilibrium phase). A homogenous mass (a: arrow) is present in the tail of the pancreas. The mass shows heterogeneously marked enhancement in the arterial phase (b: arrow) and washout in the portal venous and equilibrium phases (c, d: arrow). The splenic invasion is clearly identified in the arterial phase (b: arrowhead).

**Figure 2.**
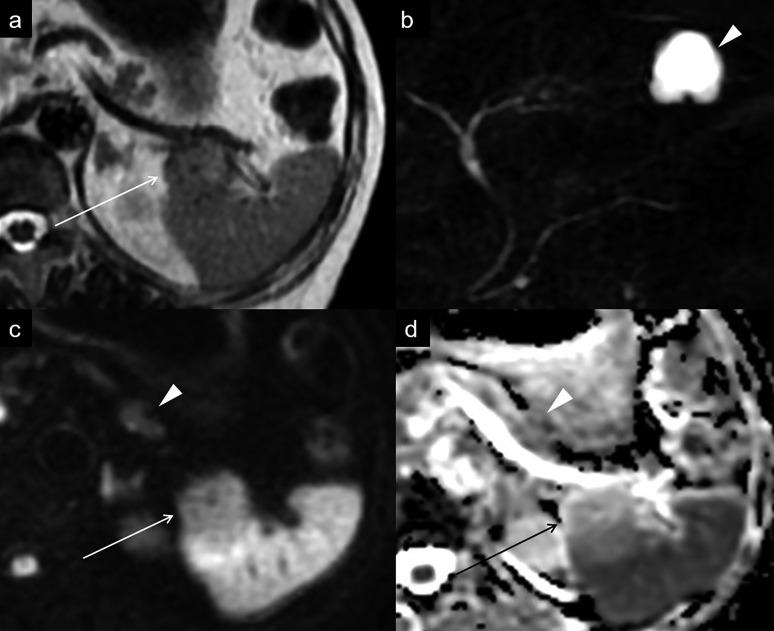
(a) On a *T*_2_ weighted image, the mass shows slightly high signal intensity without cystic components (arrow). (b) On MR cholangiopancreatography, no signal of the lesion is detected. The arrowhead shows a hepatic cyst. (c) On a diffusion-weighted image at *b-*value of 800 s/mm^2^, the mass (arrow) shows isosignal intensity compared with the pancreatic parenchyma (arrowhead). (d) ADC map shows that the mass (arrow) has higher ADC values than the pancreatic parenchyma (arrowhead). ADC, apparent diffusion coefficient.

Endoscopic ultrasonography revealed a homogenous hypoechoic mass in the pancreatic tail. Honeycomb structure was not observed. Dynamic contrast-enhanced CT revealed a 3 cm lobulated mass in the tail of the pancreas (Figure 1). The mass was almost isoattenuating to the pancreatic parenchyma and spleen in the unenhanced phase and showed heterogeneously marked enhancement in the arterial phase, with similar enhancement to the spleen in the portal venous phase and the equilibrium phase. The CT attenuation values of the mass were 40, 262, 170, and 125 Hounsfield unit (HU) for the unenhanced phase, arterial phase, portal venous phase, and equilibrium phase, respectively. Furthermore, the direct tumour invasion of the spleen was clearly visualised in the arterial phase (Figure 1b). The splenic artery and vein were not encased by the mass. No metastasis or lymph node swelling were detected. On *T*_2_ weighted MRI, the mass in the tail of the pancreas showed slightly high signal intensity, compared with the pancreatic parenchyma and spleen (Figure 2). On magnetic resonance cholangiopancreatography, neither cystic components nor dilatation of the main pancreatic duct was detected. On diffusion-weighted images with a *b*﻿-value of 800 s/mm^2^, the mass showed isosignal intensity compared with the pancreatic parenchyma. Additionally, the mass had higher apparent diffusion coefficient (ADC) values (1.63 × 10^−3^ mm^2^/s) than the pancreatic parenchyma (1.40 × 10^−3^ mm^2^/s). Based on these imaging findings, we suspected a pancreatic neuroendocrine tumour (pNET) with splenic invasion.

## Treatment/outcome/follow-up

**Figure 3.**
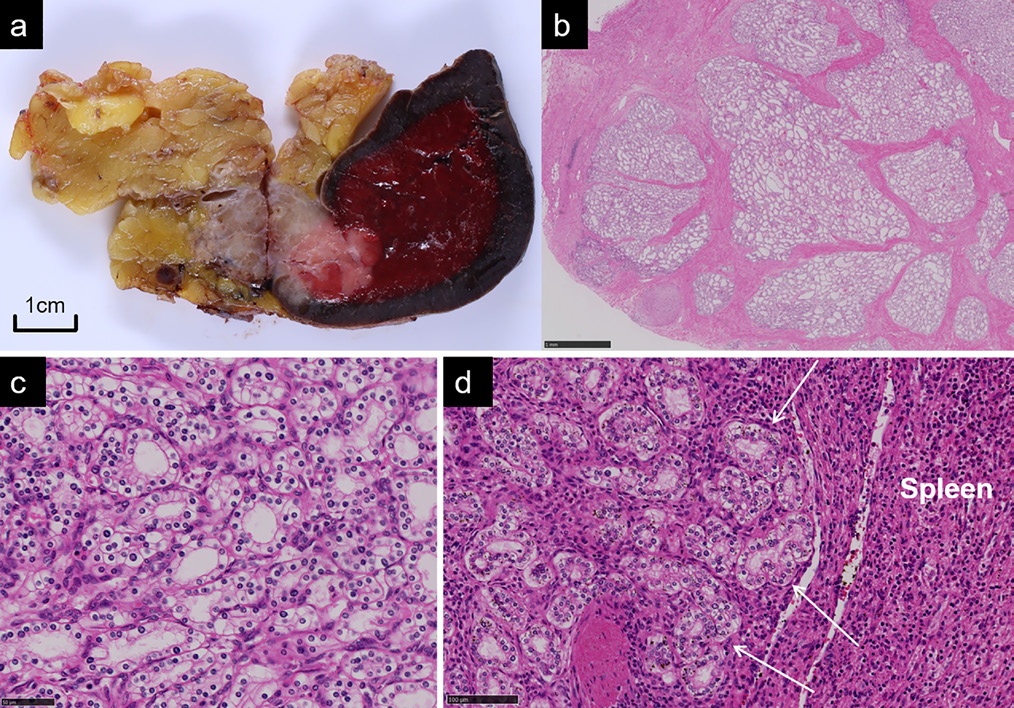
(a) Formalin-fixed specimen demonstrates a well-circumscribed tumour in the tail of the pancreas with an infiltrative growth into the spleen. Cysts are barely identifiable in the tumour. (b, c, d) Histopathological photomicrographs with hematoxylin and eosin staining shows microcystic appearance lined by epithelial cells with clear cytoplasm (b. low power field, c. high power field) and direct invasion into the splenic parenchyma (d. low power field).

Laparoscopic distal pancreatectomy with splenectomy was performed. Macroscopically, a well-circumscribed tan-pink tumour, measuring 26 × 22 × 28 mm in size, was located in the tail of the pancreas. The tumour directly invaded the spleen (Figure 3a). Cysts could barely be identified in the tumour. Microscopically, the tumour was composed of numerous tiny cysts lined by a single layer of cuboidal epithelial cells with clear cytoplasm and centrally located nuclei that were round to slightly oval (Figure 3b and c). These contained numerous glycogens, demonstrated by periodic acid-Schiff stain with and without diastase digestion. No intracytoplasmic or intraluminal mucin was observed. No mitoses or cellular atypia were noted. Ki-67 proliferation index was less than 5% in the tumour cells, indicating a low proliferation rate. Vimentin, synaptophysin, and chromogranin were not expressed; α-inhibin and MUC6 were expressed. Pathologically, there were no signs of malignancy. The lesion showed an infiltrative growth pattern into the splenic parenchyma (Figure 3d), one splenic hilar lymph node, and splenic vein. The final pathologic diagnosis was microcystic serous cystadenoma with locally aggressive features. On the seventh day post-operation, the patient was discharged without complications. After surgery, she maintained blood glucose as close to normal levels with antidiabetic drugs. She has remained alive without metastasis or local recurrence for 18 months after surgery.

## Discussion

Serous cystic neoplasms (SCNs) are relatively uncommon, accounting for 1–2% of all pancreatic neoplasms, and were first reported by Compagno^1^ and Hodgkinson^2^ in 1978. Approximately 75% of SCNs occur in females ranging between 26 and 89 years of age (mean age, 62.1 years).^3^ Almost 60% of SCNs are seen in the body or tail of the pancreas. Since patients are typically asymptomatic (61%), SCNs are usually identified incidentally.^4^

SCNs are morphologically classified into four types: microcystic, macrocystic, solid, and mixed type.^4^ The majority of cases (45%) have a macroscopic morphology characterised by innumerable small cysts, each typically less than 2 cm in diameter (microcystic type).^4^ The cysts are generally arranged in a honeycomb pattern, with thin internal enhancing septations which show low signal on *T*_2_ weighted images.^5^ A fibrous central scar with or without a stellate pattern of calcifications is detected in approximately 30% of cases.^5^ The central scar shows low signal intensity on *T*_2_ weighted images.^5^ Macrocystic type, which is the second most common type (32%), is composed of a few large cysts, each more than 2 cm in diameter.^4^

Solid type SCN has a solid gross appearance, and the cells are arranged in small acini with no or minute central lumina.^6^ The incidence of solid type was reported to be only 5% of SCNs.^4^ Compared with the microcystic type, the solid type has a homogenous appearance and is formed by cysts that are so small that the cystic structures cannot be macroscopically identified. The smaller the size becomes of each cystic component, the more difficult it is to identify them on imaging examinations. Therefore, solid type is often misdiagnosed as other solid hypervascular tumours, including pNET and metastatic renal cell carcinoma.^7^ Prior to the final diagnosis of microcystic serous cystadenoma, we preoperatively misdiagnosed the current case as pNET because the pancreatic tail mass appeared as a solid tumour with hypervascularity on dynamic CT and MRI. Based on the imaging and macroscopic findings, the tumour type was almost similar to solid type.

According to the current WHO classification, only a case with unequivocal distant metastasis beyond the pancreatic/peripancreatic bed is defined as a malignant SCN (*i.e.* serous cystadenocarcinoma), regardless of nuclear atypia or local invasion.^6^ Galanis et al reported that the malignant potential of SCNs was low (2/158 cases).^3^ In the present case, the tumour was considered as benign since no distant metastasis was detected. However, distant metastasis might be metachronous (up to 10 years) in serous pancreatic tumours,^3^ which suggests that our patient will need a long follow-up period. Even in malignant cases, death is quite rare (only one reported case of malignant SCN has reportedly resulted in death).^8^ Serous cystadenomas have also been reported to invade various organs and tissues, including the duodenum, vessels, nerves, stomach, adrenal gland, spleen, and/or colon.^9^ None of these cases were metastatic to the liver or other distant organs, and none of the patients died; these patients were predominantly female (F:M, 2.17:1), with mean age of 55 years, which is younger than that of non-invasive serous cystadenomas.^9^ In a multivariate analysis of 257 patients who underwent surgical resection of SCNs, tumour diameter and location of the tumour in the pancreatic head were independently associated with aggressive behaviour. The mean diameter of aggressive tumours was 10.5 cm (range, 3.5–27 cm).^10^ In a few cases, the relatively small tumours were reported to invade the surrounding tissues such as a 2.5 cm tumour with vascular and perivascular invasion,^11^ a 3.5 cm tumour with large vessel invasion,^10^ a 4 cm tumour with lymph node invasion,^10^ and a 4 cm tumour with neural and lymph node invasion.^12^

Only eight cases of serous cystadenomas with splenic invasion have been reported in English-language literature (Table 1).^9,10,13–18^ The diameters of all of these masses were more than approximately 9 cm, and all eight cases were symptomatic (*e.g.* abdominal pain, palpable mass, and melena). However, in the present case, a relatively small pancreatic mass (3 cm in diameter) was detected in an asymptomatic patient. To the best of our knowledge, this is the first case of incidentally detected serous cystadenoma with splenic invasion, reported with detailed imaging findings on dynamic CT and MRI.

**Table 1.** Characteristics of serous cystadenoma with splenic invasion

Author	Age (y)	Symptoms	Gross appearance	Pre-operative diagnosis	Invasion/metastasis	Tumour size (cm)	Follow-up (mo)
George et al^13^	70	Haemorrhage	Microcystic	Malignant tumour	Spleen, stomach, liver metastasis	11	Operative death
Friebe et al^14^	80	Abdominal pain	Microcystic	Pancreatic cancer or GIST	Spleen	8.9	12
Matsumoto et al^15^	87	Palpable mass	Microcystic	Malignant SCN	Spleen, large vessel, lymph node	12	10
Shintaku et al^16^	85	Fatigue, diarrhoea	Microcystic	MCN	Spleen, perineural, colon, adrenal gland	12	10
Khashab et al^10^	69	Palpable mass	NA	NA	Spleen, stomach, large vessel	17	NA
Cho et al^17^	64	Melena	Microcystic	GIST	Spleen, colon	12	12
Reid et al^9^	64	Melena, dizziness	NA	GIST	Spleen, colon	12	18
Kadhirvel et al^18^	65	Abdominal pain	Microcystic	SPN	Spleen	14	NA
**Present case**	**60** s	**None**	**Microcystic**	**pNET**	**Spleen,** **large vessel,** **lymph node**	**3.5**	**12**

GIST, gastrointestinal stromal tumour; SCN, serous cystic neoplasm; MCN, mucinous cystic neoplasm; NA, not available; SPN, solid pseudopapillary neoplasm; pNET, pancreatic neuroendocrine tumour

In conclusion, the current report presents a rare case of microcystic serous cystadenoma with splenic invasion, with a small mass that was detected incidentally. It is important to recognise that pancreatic serous cystadenomas can show locally aggressive features, even if they are small in size, and that they have a limited malignant potential with a favourable prognosis.

## Learning points

1. Serous cystadenomas of the pancreas can show invasive growth into the surrounding tissues, even if they are small in size.
2. Serous cystadenomas of the pancreas with locally aggressive features tend to have a favourable prognosis, according to previous reports.
